# Central and Peripheral Signals Set the Circadian Liver Clock

**DOI:** 10.1371/journal.pbio.0050050

**Published:** 2007-01-30

**Authors:** Liza Gross

The importance of maintaining a smooth-running circadian clock becomes painfully evident whenever we suffer severe jet lag. Traveling through multiple time zones decouples our biological rhythms from the natural cycle of light and dark we’re used to. These light–dark cycles synchronize everything from basic metabolic processes to feeding behavior. People with total blindness, who cannot perceive these light cues because they lack functional retinal photoreceptors, have disrupted circadian rhythms. As mammals, we perceive light cues through retinal photoreceptors that relay the signals to a cluster of some 16,000 neurons in the hypothalamus called the suprachiasmatic nucleus (SCN).

The Greenwich Mean Time of the circadian system, the SCN sets the cycle of the circadian clocks found in nearly every cell in the body. “Phase adjustments” in peripheral tissues make sure each clock follows the same schedule. Phase adjustments can be set indirectly, through biological rhythms that are themselves SCN-dependent, such as feeding cycles or body temperature. How these synchronizing cues operate at the molecular level remains obscure. Of particular interest is whether cyclically expressed genes in peripheral tissues are controlled by local circadian clocks or by systemic cues that are directly or indirectly controlled by the SCN pacemaker.

In a new study, Beno
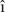
t Kornmann, Ueli Schibler, and colleagues investigated this question by focusing on circadian genes in the liver. To tell whether genes were under local or systemic control, they needed to shut down the local circadian oscillators (the molecular mechanisms responsible for cyclical gene expression) in the liver. They solved that problem by generating a mouse strain with conditionally active liver clocks that they could turn on or off at will.

To create their model, the authors modified a transcription factor called REV-ERBα that shuts down circadian gene expression by suppressing the core clock gene *Bmal1*. The modified gene harbored regulatory elements, called tetracycline-responsive elements (TREs), that respond to tetracycline and similar antibiotics. A mouse strain with this modified gene was bred with a strain carrying a gene construct that activates the elements specifically in liver cells. When the transgenic offspring, which carried both transgenes, ate the tetracycline-like drug doxycyline (Dox) in their food, *Rev-erbα* remained silent in liver cells and the circadian oscillator functioned normally. In the absence of Dox, transgenic offspring overexpressed REV-ERBα, and *Bmal1* remained inactive throughout the day, effectively turning off the liver clocks.

When the authors analyzed the expression of genes targeted by BMAL1 in the presence and absence of Dox, they found arrhythmic expression of most of the targeted clock genes when the liver clocks stopped, as expected. But they were surprised to discover that this was not the case for the core clock gene *mPer2*: rhythmic expression of *mPer2* transcript and protein levels continued even in the absence of BMAL1 activity. This result is particularly noteworthy because in brain tissue from transgenic mice lacking *Bmal1*, *mPer2* transcripts in SCN neurons are barely noticeable during the course of a day.

**Figure:**
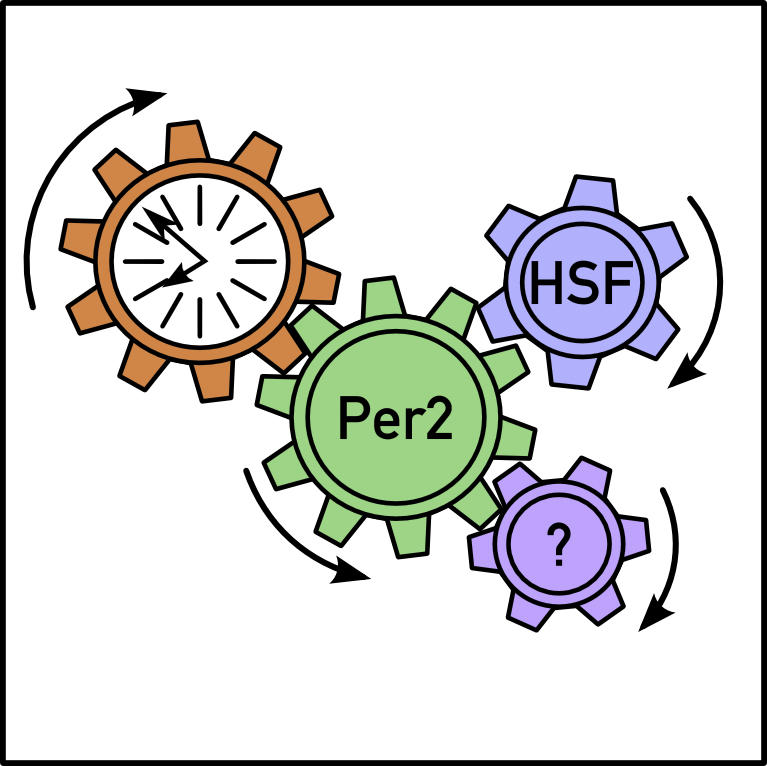
System-driven circadian gene transcription provides a link to synchronize the phase of the peripheral oscillator to that of the central system.

While it’s possible that *mPer2* transcription is more dependent on BMAL1 in the SCN than it is in the liver, the authors considered two other explanations for the observed *mPer2* activity: either the mice that didn’t eat Dox still harbored enough BMAL1 to drive cyclic *mPer2* expression or oscillating systemic signals were responsible. To distinguish between these possibilities, they analyzed temporal *mPer2* expression in isolation from master pacemaker signals by removing liver slices from mice carrying a fluorescent protein attached to *mPer2*. With this approach, luminescence signals showed a rhythmic profile that follows *mPer2* activity.

The authors bred the mouse strain with the conditionally active clock (which needs Dox for BMAL1 activation) with a strain carrying the *mPer2* fusion gene (which glows in response to the light-emitting molecule luciferin). Liver slices from the offspring of these mice did not generate circadian expression of fluorescent *mPer2* products in the absence of Dox, indicating that the liver oscillators had been shut off. When Dox was added to the medium supporting the liver tissue, circadian luminescence rhythms matched those seen in livers from mice with fluorescent *mPer2* and unimpeded *Bmal1* expression. The patterns of *mPer2* expression seen in the liver explant experiments together with those seen in livers taken from the transgenic offspring show that liver oscillators can drive circadian gene expression in the absence of systemic cues and that systemic cues can do so in the absence of liver oscillators.

A genome-wide scan of locally and systemically regulated circadian genes identified 351 transcripts in animals fed Dox-laced chow. Cyclic expression of most of these genes requires a functional liver clock, the authors concluded, because their circadian amplitude dropped significantly in mice that ate Dox-less chow. Over 30 genes were also expressed in animals on the normal diet, including *mPer2* and a number of heat-shock proteins (HSPs). The heat-shock and *mPer2* genes followed the same arc and temporal patterns, suggesting the influence of a shared systemic cue—which the authors reveal as elevated temperature. Since *mPer2* transcription can be induced by heat shock and since the *mPer2* gene contains binding sites for the transcription factors (HSFs) that activate heat-shock proteins, the authors plan to investigate in future experiments whether these transcription factors and binding sites help mediate the temperature-dependent expression of *mPer2*.

By controlling liver clock function with the flip of a molecular switch, the authors showed that only 10% of circadian genes follow temporal expression patterns in the absence of liver oscillators. These genes likely respond to physical or chemical signals that are controlled by the master SCN pacemaker, the authors argue. *mPer2* is a strong candidate as one of the systemically regulated synchronizers because its expression pattern is nearly identical with or without a functional liver clock. A systemically driven *mPer2* transcription cycle would provide the necessary link to synchronize the phase of the peripheral oscillator to that of the central system.

Future studies are necessary to illuminate the molecular mechanisms of systemic *mPer2* transcription. A number of environmental cues—including elevated temperature and oxidative stress—activate HSF1. It’s possible that HSF1, by sensing rhythmic changes in metabolism, synchronizes peripheral clocks by modulating *mPer2* expression. These and similar questions about the signaling pathways that bring all the body’s clocks into the same phase can be explored with the novel transgenic mouse model described here.

